# Intrathecal morphine in combination with bupivacaine as pre-emptive analgesia in posterior lumbar fusion surgery: a retrospective cohort study

**DOI:** 10.1186/s13018-022-03124-2

**Published:** 2022-04-18

**Authors:** R. Trivedi, J. John, A. Ghodke, J. Trivedi, S. Munigangaiah, S. Dheerendra, B. Balain, M. Ockendon, J. Kuiper

**Affiliations:** The Robert Jones and Agnes Hunt Orthopaedic Hospital, Gobowen, Oswestry, SY10 7AG UK

**Keywords:** Posterior lumbar fusion surgery, Intrathecal morphine, Spinal anaesthesia, Opioids, Enhanced recovery after surgery

## Abstract

**Background:**

The purpose of this study was to evaluate the efficacy of intrathecal morphine (ITM) in combination with bupivacaine as pre-emptive analgesia in patients undergoing posterior lumbar fusion surgery. This is in comparison with traditional opioid analgesics such as intravenous (IV) morphine.

**Methods:**

Two groups were identified retrospectively. The first (ITM group) included patients who had general anaesthesia (GA) with low-dose spinal anaesthesia prior to induction using 1–4 mls of 0.25% bupivacaine and 0.2 mg ITM. 1 ml of 0.25% bupivacaine was administered per hour of predicted surgery time, up to a maximum of 4 ml. The insertion level for the spinal anaesthetic corresponded to the spinal level of the iliac crest line and the level at which the spinal cord terminated. The control group had GA without any spinal anaesthesia. Patients were instead administered opioid analgesia in the form of IV morphine or diamorphine. The primary outcome was the consumption of opioids administered intraoperatively and in recovery, and over the first 48 h following discharge from the post-anaesthesia care unit (PACU). Total opioid dose was measured, and a morphine equivalent dose was calculated. Secondary outcomes included visual analogue scale (VAS) pain scores in recovery and at day two postoperatively, and the length of stay in hospital.

**Results:**

For the ITM group, the median total amount of IV morphine equivalent administered intraoperatively and in recovery, was 0 mg versus 17 mg. The median total amount morphine equivalent, administered over the first 48 h following discharge from PACU was 20 mg versus 80 mg. Both are in comparison with the control group. The median length of stay was over 1 day less and the median VAS for pain in recovery was 6 points lower. No evidence was found for a difference in the worst VAS for pain at day two postoperatively.

**Conclusion:**

ITM in combination with bupivacaine results in a significantly decreased use of perioperative opioids. In addition, length of hospital stay is reduced and so too is patient perceived pain intensity.

*Trial registration* The study was approved by the ethics committee at The Robert Jones and Agnes Hunt Orthopaedic Hospital as a service improvement project (Approval no. 1617_004).

## Background

Orthopaedic surgeons are known to prescribe the third-highest number of opioid prescriptions amongst all specialities [1]. Moreover, spine surgery is thought to carry a higher risk of both preoperative and long-term post-operative use due to the painful nature of surgery and the often-chronic course of the underlying condition [2]. Perhaps most strikingly, a study characterising the risk of long-term opioid use in lumbar spinal surgery demonstrated that 7.5% of opioid naïve patients became opioid dependent post-surgery [2].

However, surgeons and anaesthetists have consistently endeavoured to tackle this problem. In particular, enhanced recovery after surgery (ERAS) bundles are used to reduce patient morbidity post-surgery [3]. ERAS pathways, which typically incorporate a standardised, multimodal analgesic (MMA) regimen with nonopioid agents, spinal anaesthesia and regional blocks have been proven to control pain after orthopaedic surgery [3].

To date, there have been a limited number of reports that implement ERAS bundles that focus on improving patient outcomes in lumbar surgery [3]. Posterior lumbar fusion surgery is a highly painful procedure and usually requires significant amounts of opioid for adequate perioperative analgesia. As widely documented, the use of high dose opioids can commonly be associated with adverse effects, including opioid-induced hyperalgesia, nausea, ileus, and even respiratory depression.

The use of ITM on its own, has consistently been reported to provide analgesia after major spinal surgery [4]. However, ITM in combination with a local anesthetic is an emerging technique that, when implemented within an ERAS pathway, has the potential to significantly minimize opioid requirements [5]. The authors of this study hypothesized that using ITM and bupivacaine, in combination with GA will reduce perioperative opioid requirements enabling faster discharge from recovery, earlier mobilization and hence a shorter length of stay. The objective was to compare this type of pre-emptive analgesia with traditional perioperative opioid analgesics such as intravenous (IV) morphine.

## Methods

### Inclusion and exclusion criteria

Patients of all ages, with an ASA score of 1–3, who had posterior lumbar fusion surgery between 1 January 2015 and 31 December 2016, at a tertiary spinal centre were retrospectively studied. Informed consent was obtained from all patients prior to undertaking this research. During the 2-year period, two different anaesthetic techniques were routinely adopted for this type of surgery. Firstly, that of ITM in combination with local anaesthetic and GA, and secondly a standard technique that involved GA alone. The choice for ITM with local anaesthetic was anaesthetist dependent. For the patient’s included in the study, the surgical and anaesthetic teams remained consistent.

Two patient groups were identified based on anaesthetic technique. First, an ITM group and secondly, the control, who were administered GA alone. Patients on preoperative strong opioids and those not able to tolerate non-steroidal anti-inflammatories (NSAIDs) were excluded.

### ITM group

The ITM group had GA with low dose spinal anaesthesia prior to induction using 1–4 mls of 0.25% bupivacaine and 0.2 mg ITM (0.2 ml of 1 mg/ml solution). Spinal anesthetic was performed with the patient awake, prior to induction of a GA. The insertion level for the spinal anesthetic was determined following review of the patient’s MRI scan. More specifically, we determined the spinal level corresponding to the iliac crest line and the level at which the spinal cord terminated. The dose of bupivacaine was calculated as per the predicted duration of surgery. 1 ml of 0.25% bupivacaine was used per hour of predicted surgery time, up to a maximum of 4 ml. By keeping the dose of spinal anesthetic low, the assessment of motor function in recovery was feasible. GA was induced with propofol and remifentanil and maintained with sevoflurane and remifentanil. 1 g IV paracetamol and 40 mg IV parecoxib were given in theatre and postoperatively regularly, along with 0.5 mg/kg ketamine in theatre. No long-acting opioids were given in this group in theatre. Hypotension, although concurring the benefit of reducing blood loss, was a common side effect in this group. Metaraminol was administered as required, intraoperatively, to keep the mean BP > 65 mmHg.

### Control group

The control group had a similar GA, using fentanyl or remifentanil with sevoflurane. No spinal anesthetic was administered. Multimodal analgesia was used as in the ITM group, including the same dose of paracetamol, parecoxib and ketamine. Intraoperatively, patients were administered traditional peri-operative opioid analgesics in the form of IV morphine or diamorphine at the anesthetist’s discretion.

Both groups (ITM and control), had local paraspinal muscle infiltration using 60 ml 0.25% bupivacaine intra-operatively. This was administered irrespective of patient weight and the number of spinal levels operated on. No patient weighed less than 60 kg, therefore, there was no risk of overdose.

Muscle infiltration was carried out in two stages. First, 30 ml was placed bilaterally in the paraspinal muscles after dissection was complete, prior to commencing decompression. The following 30 ml was injected into the skin and subcutaneous tissue prior to closure.

Following discharge from PACU, both groups had equal access to immediate release oxycodone and IV morphine or diamorphine for pain. The dose of oxycodone and diamorphine administered was converted into a morphine equivalent and was measured over a 48-h period. Regular oral paracetamol and NSAIDs were used on the ward in both groups. No IV PCA was used in either group. All patients received 3.3 mg dexamethasone and 4 mg ondansetron as antiemetic prophylaxis. Furthermore, all patients had an indwelling urinary catheter placed postoperatively, avoiding complications of urinary retention.

Pain scores were recorded every 10 min in the immediate postoperative phase using a numerical 0–10 VAS scale. Pain scores were subsequently assessed following discharge from PACU by ward nurses on a 4 hourly basis for 48 h. These data were collected retrospectively from patient notes and drug charts.

For the two groups, we compared the following:

1. Amount of IV morphine equivalent administered intraoperatively and in recovery
2. Amount of morphine equivalent administered over the first 48 h following discharge from PACU
3. Visual analogue scale (VAS) pain scores at recovery
4. Worst VAS pain scores until postoperative day two
5. Length of stay (LOS)

Morphine equivalents were calculated according to guidelines published in NICE [6]. 6 mg IV diamorphine = 10 mg IV morphine = 13 mg oral oxycodone.

### Statistical analysis

We used a covariate-balancing propensity score weighting method to remove bias between the two groups in terms of age, gender, ASA grade and the number of fused spinal levels [7]. For the latter, we regarded the number of fused levels as continuous variables. The average treatment effects (ATE) were then estimated using a linear model with a robust (sandwich) variance estimator. An inverse probability treatment weighting (IPTW) was used to implement the propensity weights [8]. In addition, we used the standardised mean difference (SMD) to assess covariate balance and assumed that any imbalance above 10% would indicate a meaningful imbalance [8]. A *p* value below 0.05 was assumed to denote statistical significance. The required sample size was estimated based on the total morphine equivalent consumption over the first 48 h following discharge from PACU. We assumed a reduction of 50% in total morphine equivalent would be clinically relevant. A comparable study evaluating patients undergoing transforaminal interbody fusion (TLIF) surgery found a mean 48-h total morphine consumption of 82 mg and a standard deviation of 46 mg for the control group [9]. Based on these numbers, the minimum relevant effect size would be 41/46 = 0.89. In turn, we would need a minimum of 42 patients (21 in each group) to achieve 80% power at the two-tailed *p* = 0.05 significance interval. All analyses assumed that a two-tailed *p* = value below 0.05 denoted significance. Analyses were performed using R vs 3.6.1 (R Foundation for Statistical Computing, Vienna, Austria) and the packages “CBPS” and “survey”.

## Results

In all, 66 patients were identified, of whom 26 had spinal anaesthesia in the form of ITM and 0.25% bupivacaine. Most patients in both groups were female, with a slightly higher mean age in the ITM group (Table 1). In addition, the ITM group had a higher fraction of patients who were deemed ASA grade 1 and a lower fraction of patients with ASA grade 2. On average, a greater number of spinal levels were fused in the ITM group. The age and ASA grade 2 imbalances were clearly above the 10% meaningful imbalance threshold, and the gender and ASA grade 2 imbalances were borderline meaningful (Table 1). After covariance balancing, the two propensity score weighted samples were balanced with respect to all four baseline characteristics (Table 1).

**Table 1:** Baseline characteristics of the two groups and imbalance between the original (unweighted) and propensity score—weighted groups

Characteristic	Level	Spinal anaesthesia (ITM + 0.25% bupivacaine)		Propensity weighting	
		No	Yes	SMD (%; before)	SMD (%; after)
*n*		40	26		
Gender (%)	F	24 (60.0)	14 (53.8)	12.5	10.0
	M	16 (40.0)	12 (46.2)		
Age (mean (SD))		48.77 (12.82)	52.42 (13.17)	28.1	6.0
ASA grade (%)	1	19 (47.5)	14 (53.8)	12.7	7.7
	2	19 (47.5)	10 (38.5)	− 18.3	− 9.4
	3	2 (5.0)	2 (7.7)	11.1	3.3
Levels (%)	1	17 (42.5)	11 (42.3)	10.7	8.1
	2	22 (55.0)	12 (46.2)		
	3	0 (0.0)	2 (7.7)		
	4	0 (0.0)	1 (3.8)		
	5	1 (2.5)	0 (0.0)		

Propensity-score matching was based on all four baseline characteristics

Standardised mean difference (SMD), expressed as %. The number of levels was considered a continuous variable in the propensity score analysis

**Fig. 1:**
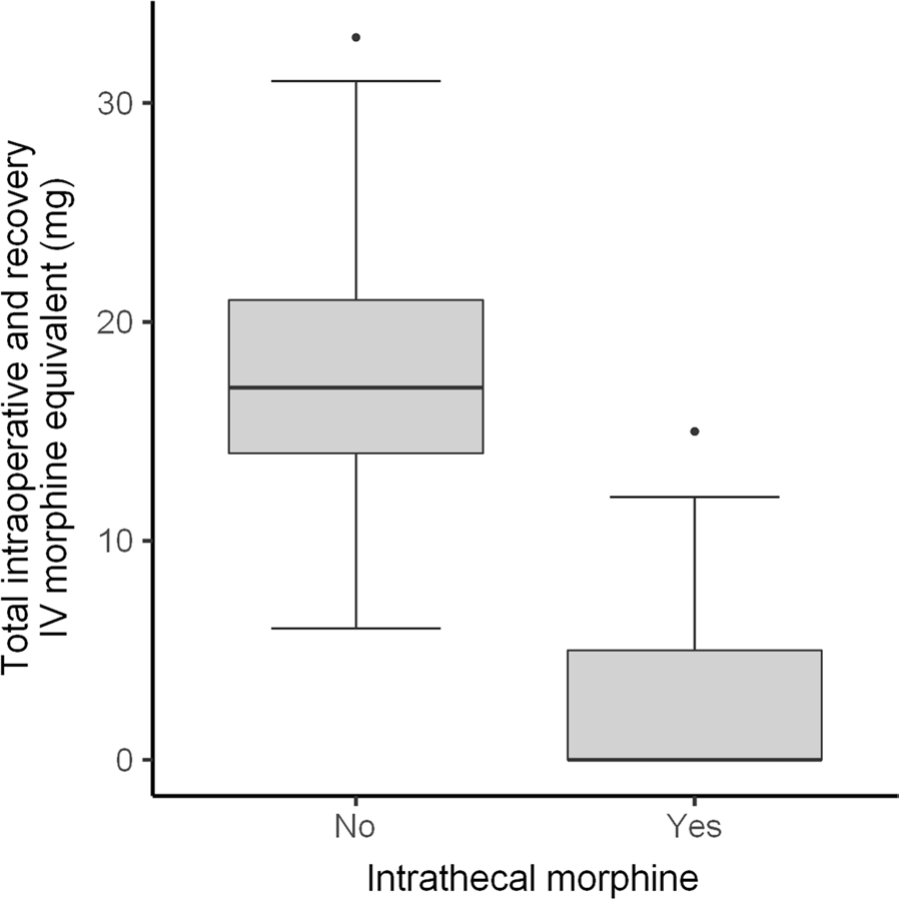
Distribution after propensity score weighting of total intraoperative and recovery IV morphine equivalent (mg) for the control group and the ITM cohort

**Fig. 2:**
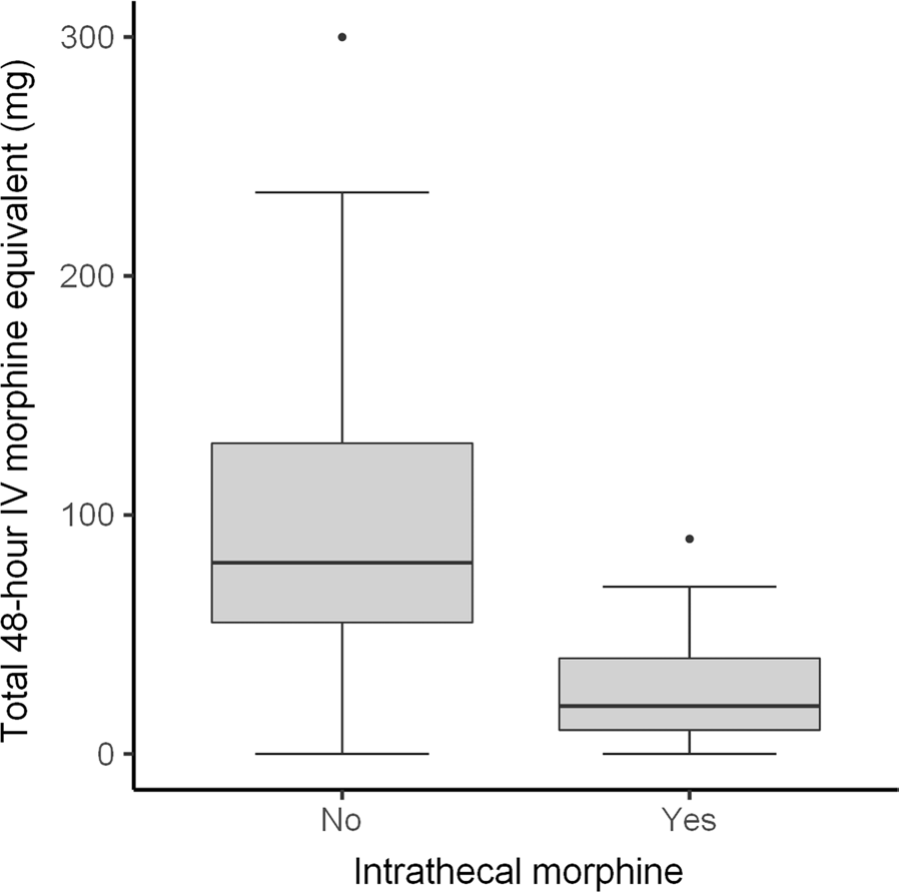
Distribution after propensity score weighting of total 48-h morphine equivalent (mg) for the control group and the ITM cohort

**Fig. 3:**
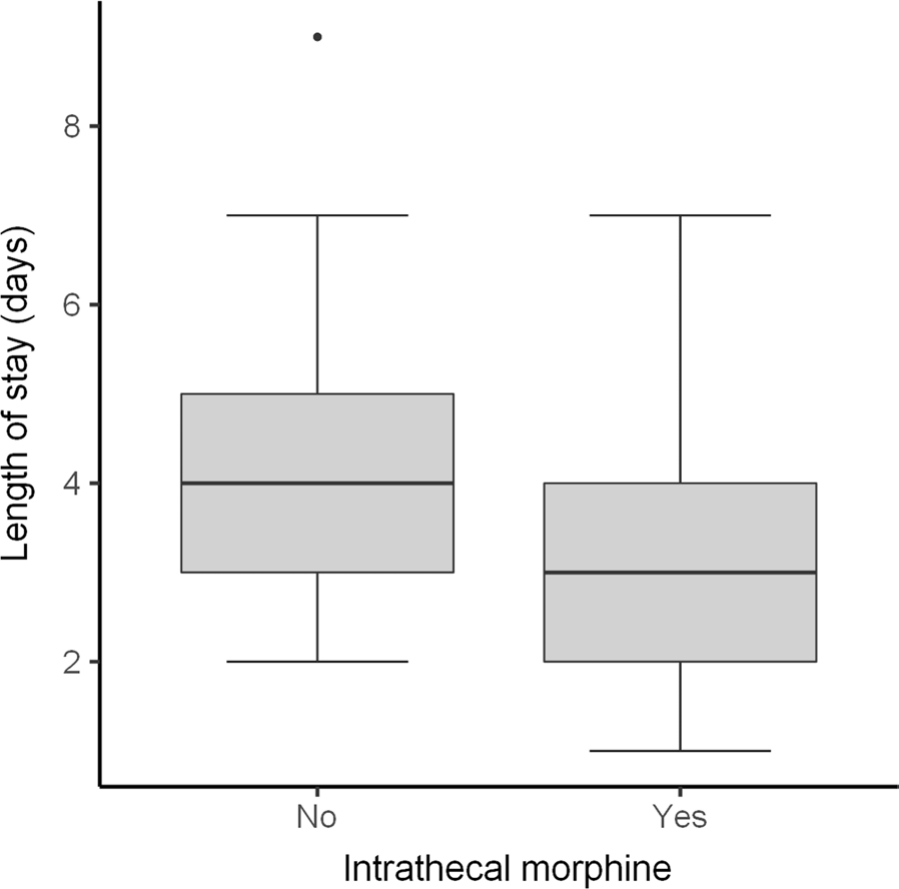
Distribution after propensity score weighting of total length of stay (in days) for the control group and the ITM cohort

**Fig. 4:**
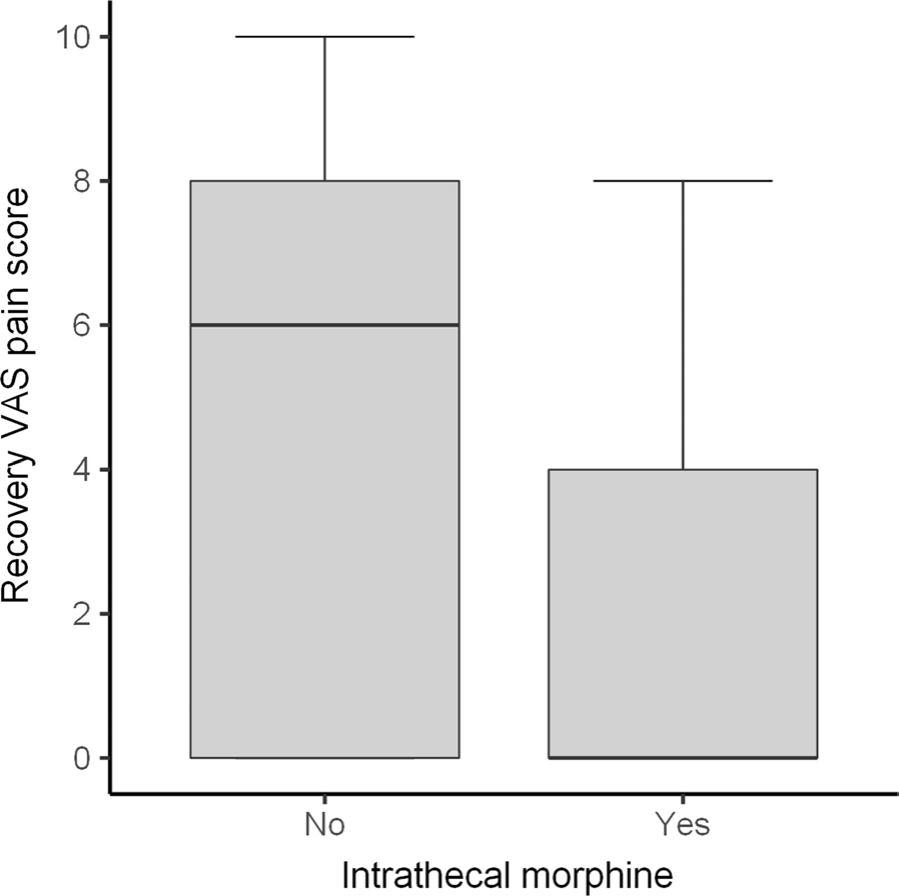
Distribution after propensity score weighting of visual analogue scale (VAS) scores for pain at recovery for the control group and the ITM cohort

**Fig. 5:**
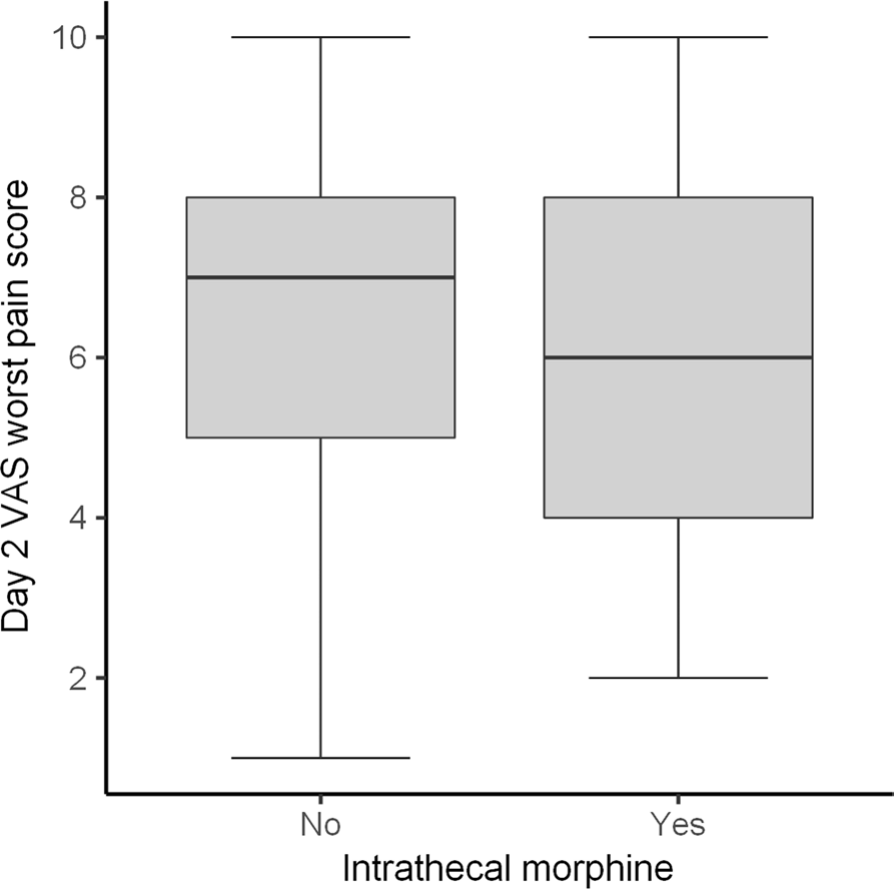
Distribution after propensity score weighting of visual analogue scale (VAS) scores for worst pain at day 2 postoperatively for the control group and the ITM cohort

A comparison of outcomes between the ITM and control group is depicted in Table 2. When considering weighted values, the median amount of IV morphine equivalent administered intraoperatively and in recovery was 17 mg less in the ITM group. In addition, the median amount morphine equivalent administered over the first 48-h following discharge from PACU was 60 mg less. (Figs. 1, 2). A greater percentage of patients (69% vs 10%) in the ITM group had either no pain or mild pain in recovery compared to the control group. The incidence of severe pain in recovery was significantly lower in the ITM group than the control group (19% vs 49%). This is despite the control group receiving more opioids in theatre. Furthermore, the median length of stay was over 1 day less in the ITM group (Fig. 3). Lastly, the median VAS for pain at recovery was 6 points lower in patients who received spinal anaesthesia with ITM and 0.25% bupivacaine (Fig. 4). No evidence was found for a difference in the worst VAS for pain at day 2 (Fig. 5).

**Table 2:** Comparison of outcomes between the ITM and control group

	Raw values (median, IQR)		Weighted values (median, IQR)		Weighted average treatment effect	
	Control group	ITM	Control group	ITM	Difference in means (95% CI)	*p* value
Total IV morphine equivalent administered intraoperatively and in recovery (mg)	18 (14–21)	0 (0–4)	17 (14–21)	0 (0–5)	15.6 (12.9–18.0)	< 0.001
Total 48-h morphine equivalent (mg)	88 (58–130)	20 (2.5–40)	80 (55–130)	20 (10–40)	75.3 (49.7–90.5)	< 0.001
Length of stay (days)	4 (3–5)	3 (2–4)	4 (3–5)	3 (2–4)	1.3 (0.5–2.1)	0.006
VAS pain recovery (0–10)	6 (0–8)	0 (0–4)	6 (0–8)	0 (0–4)	2.8 (1.3–4.7)	0.001
Worst VAS pain day 2 (0–10)	7 (5–8)	6 (4–8)	7 (5–8)	6 (4–8)	0.9 (-0.4–2.0)	0.19

The weighted average treatment effect was calculated based on propensity score weighting

We examined patient notes for recordings of common side effects of opioid use. Whilst no patient had respiratory depression as evidenced by recordings of respiratory rate and oxygen saturations, documentation of side effects such as pruritis were inconsistent. Nausea scores and antiemetic requirements were recorded and showed no difference between the two groups.

## Discussion

This study demonstrates that low dose spinal anaesthetic using 0.2 mg ITM and 0.25% bupivacaine, combined with GA and surgical site local anaesthetic infiltration significantly reduces perioperative opioid requirements. This enables faster discharge from recovery and hence a shorter length of stay.

Posterior lumbar surgery is a highly invasive procedure, resulting in significant soft tissue and muscle dissection. Pain control regimens following this type of surgery have consistently relied on opioid medications. Although beneficial in the management of severe acute post-operative pain, opioids have been linked with adverse effects such as increased wound complications, respiratory depression, nausea and vomiting and constipation [5]. These side effects significantly contribute to the morbidity of spinal surgery. Jain et al. investigated the correlation between opioid therapy and post-operative outcomes following posterior lumbar fusion surgery [10]. Patients in the opioid cohort had a 1.2-fold increase in the risk for all complications, including new pain diagnoses and emergency department visits within 90 days of surgery [10]. In conjunction, Lee et al. examined the correlation between opioid consumption and post-operative function as measured by patient-reported outcome measures [11]. For every 10 mg increase in daily morphine equivalent there was an associated 0.03 decrease in the 12-Item Short-Form Health Survey physical component summary and mental component summary score, a 0.01 decrease in the EuroQol-5D score, and a 0.5 increase in the Oswestry Disability Index and Neck Disability Index score at twelve months postoperatively [11]. This was proven in 583 patients undergoing spinal surgery.

Opioid reduced anaesthesia offers a tool for minimizing opioid use and their side effects, thereby improving outcomes following spinal surgery. O’Neill et al. described the first report on the use of ITM for relief of postoperative pain after lumbar spine surgery [12]. They demonstrated that 46% of those treated did not require any additional analgesia [12]. In concurrence, a meta-analysis looking at the efficacy of ITM showed that there was a significant reduction in pain scores and opioid consumption in the first post-operative day [4]. Eight studies were included in this analysis, however, heterogeneity existed in the fact that each dose of ITM varied considerably between trials [4]. Our study demonstrated a significant effect in outcomes using a dose of 0.2 mg of ITM. However, Yörükoglu et al. concluded that a dose of 0.1 mg ITM also resulted in a significant reduction in early post-operative analgesic requirement with insignificant side effects [13]. A significant difference between our study and those included in the meta-analysis is that we administered morphine in the operating theatre before the surgery along with local anesthetic. Most other studies administered morphine before wound closure under direct visualization of the intrathecal space. The benefit of the former means the sensory block produced by the local anesthetic provides intense pre-emptive analgesia, while the intrathecal morphine has an onset of action at 1 h and a peak at 3 h. Similarly, Wang et al. described the efficacy of pre-emptive analgesia in multilevel posterior lumbar interbody fusion surgery [14]. They also used 0.2 mg of ITM and compared with the control group, the ITM group had a significantly reduced consumption of intraoperative remifentanil, postoperative sufentanil and supplemental analgesics [14]. Furthermore, regarding patient comfort, the ITM group had a greater degree of satisfaction with the whole hospitalisation experience. This was in comparison with the control group, who received 2 ml of 0.9% saline prior to anaesthesia induction [14]. A significant difference between our study at that conducted by Wang et al. is that we were able to directly measure VAS scores within the first hour postoperatively in recovery and demonstrate a significant effect.

With regards to post-operative side-effects of ITM, pruritis has been reported in multiple large-scale studies [15, 16]. However, a continuous infusion of low dose naloxone has been recommended as a treatment for opioid-induced pruritis [17]. Another, more serious side-effect is delayed-onset respiratory depression due to the gradual spread of morphine through cerebrospinal fluid following injection. Nonetheless, in the aforementioned meta-analysis, the incidence of respiratory depression was only 2.6% in the ITM group [4]. Furthermore, the incidence was only estimable in two studies, both of which administered doses of ITM far greater than 0.2 mg [4]. In our study, there were no cases of morphine-associated respiratory depression in the first 48 h postoperatively.

The current clinical study used ITM in combination with bupivacaine. The synergism between local anaesthetics and opioids in anaesthesia is a concept that has been successfully used for many years [18]. Tejwani et al. suggested that bupivacaine induces conformational change in the spinal opioid receptor thereby expediting this synergism [18]. It is also widely accepted that a combination of morphine and bupivacaine is more effective than either of them alone in producing effective pain relief [18, 19]. In addition to good analgesia, Bachmann et al. demonstrated that bupivacaine used in combination with ITM caused less motor block than a higher dose of bupivacaine alone in patients undergoing hip and knee arthroplasty [18]. This is particularly important in orthopaedic surgery, where patients are encouraged to mobilize as early as possible following surgery to facilitate recovery. Another advantage of using a combination of a spinal with GA is hypotensive anaesthesia. Deliberate hypotension during anaesthesia for major spinal surgery concurs the benefit of reducing blood loss and thereby transfusion requirement [20]. The traditional approach to achieving hypotension involves an overdose of the anaesthetic or a cardiac depressant drug. In the presence of a spinal, the blood pressure falls without increasing the anaesthetic depth and the need for cardiac depressant drugs. This facilitates a faster recovery.

To our knowledge, this present study is the first to demonstrate the effective use of ITM in combination with bupivacaine in posterior lumbar fusion surgery. However, our study had some limitations. First, we evaluated only a single low dose of ITM. Additional studies are required to demonstrate analgesic efficacy at different doses of ITM. Second, despite opioid side effects such as nausea and vomiting being assessed, others such as pruritis were inconsistently recorded and therefore not evaluated. Third, due to the retrospective nature of the study and an inability to randomize patients, a degree of selection and treatment bias was inevitable. Furthermore, a long-term follow-up of patients requiring opioids after discharge was not included in this analysis. Despite these limitations, we believe this study addressed its primary objective to demonstrate the efficacy of low-dose ITM in combination with bupivacaine in reducing perioperative opioid consumption in posterior lumbar fusion surgery.

## Conclusions

In conclusion, our study shows, that for patients undergoing posterior lumbar fusion surgery, 0.2 mg of ITM in combination with 0.25% bupivacaine, prior to the induction of GA results in significantly preferable outcomes. Perioperative opioid requirements were reduced, length of stay was considerably shorter and VAS pain scores at recovery were lower. Therefore, the application of this protocol in practice settings should be considered, particularly within ERAS bundles that promote patient safety and better clinical outcomes.

## Data Availability

All data generated or analysed during this study are included in this published article.
